# Skeletal Muscle Metabolism Is Dynamic during Porcine Postnatal Growth

**DOI:** 10.3390/metabo14070357

**Published:** 2024-06-26

**Authors:** Linnea A. Rimmer, Erika R. Geisbrecht, Michael D. Chao, Travis G. O’Quinn, Jason C. Woodworth, Morgan D. Zumbaugh

**Affiliations:** 1 Department of Animal Sciences and Industry, Kansas State University, Manhattan, KS 66506, USAtravisoquinn@ksu.edu (T.G.O.);; 2 Department of Biochemistry and Molecular Biophysics, Kansas State University, Manhattan, KS 66506, USA

**Keywords:** skeletal muscle metabolism, postnatal growth, mitochondria, swine

## Abstract

Skeletal muscle metabolism has implications for swine feed efficiency (FE); however, it remains unclear if the metabolic profile of skeletal muscle changes during postnatal growth. To assess the metabolic changes, samples were collected from the *longissimus dorsi* (LD, glycolytic muscle), *latissimus dorsi* (LAT, mixed muscle), and *masseter* (MS, oxidative muscle) at 20, 53, 87, 120, and 180 days of age from barrows. Muscles were assessed to determine the abundance of several metabolic enzymes. Lactate dehydrogenase (LDHα) decreased in all muscles from 20 to 87 d (*p* < 0.01), which may be attributed to the muscles being more glycolytic at weaning from a milk-based diet. Pyruvate carboxylase (PC) increased in all muscles at 53 d compared to the other time points (*p* < 0.01), while pyruvate dehydrogenase α 1 (PDHα1) increased at 87 and 180 d in MS compared to LD (*p* < 0.05), indicating that potential changes occur in pyruvate entry into the tricarboxylic acid (TCA) cycle during growth. Isolated mitochondria from each muscle were incubated with ^13^C-labeled metabolites to assess isotopomer enrichment patterns of TCA intermediates. Citrate M + 2 and M + 4 derived from [^13^C_3_]-pyruvate increased at 87 d in LAT and MS mitochondria compared to LD mitochondria (*p* < 0.05). Regardless of the muscle, citrate M+3 increased at 87 d compared to 20, 53, and 120 d, while 180 d showed intermediate values (*p* < 0.01). These data support the notion that pyruvate metabolism is dynamic during growth. Our findings establish a metabolic fingerprint associated with postnatal muscle hypertrophy.

## 1. Introduction

The U.S. produces 11% of the world’s pork, which is the most widely consumed meat globally, with U.S. exports valued at over USD 7.7 billion [1]. As the global population continues to increase and the demand for lean meat rises, pork production in the U.S. is predicted to grow by 2.2 billion pounds per year from 2023 to 2031 [2]. Over the same period, the planted acreage of the eight major row crops is predicted to decrease by 3.9 million acres [2], which depicts the need for innovative strategies to increase swine production with less land and resources. Although market pigs in the U.S. today require 4% less feed and have a 17% heavier carcass than those reared three decades ago [3], additional improvements are becoming more difficult to obtain. For example, feed conversion ratios decreased by 44% on feeder-to-finish operations from 1992 to 2004 but only decreased by 3% from 2004 to 2009 [4]. In addition to reducing feed requirements, the consumer preference for leaner pork also requires strategies to produce heavier, muscled pigs [5,6]. While traditional production strategies are unarguably imperative for continued advancements, innovative strategies to further improve production performance are necessary to further increase lean pork production with less arable land.

Skeletal muscle is an energetically demanding tissue that accounts for 40–50% of body mass [7,8]. Muscle is composed of a heterogenous population of muscle fibers that are classified by contractile speed (slow-twitch or fast-twitch) and metabolism (glycolytic or oxidative). Slow-twitch muscle fibers rely on oxidative metabolism, while fast-twitch muscle metabolism can range from highly oxidative to highly glycolytic [9,10]. The metabolic profile of muscle has significant impacts on global nutrient utilization efficiency. For example, the skeletal muscle of pigs with improved FE upregulates glycolytic enzymes compared to low FE pigs [11]. Further, skeletal muscle mitochondrial electron leak is negatively associated with FE in cattle and broilers [12,13]. Although it is apparent that the metabolic characteristics of skeletal muscle influence the efficiency of animal growth, the complex dynamics of postnatal growth presumably involves changes in skeletal muscle metabolism during postnatal growth.

Postnatal growth can be theoretically divided into four phases. After birth, phase I prioritizes the growth of vital organs, the nervous system, visceral fat, and bone development. Phase II supports rapid lean accretion, while phase III begins to decrease the rate of muscle hypertrophy, and phase IV promotes fat deposition [9]. Although an oversimplification, outlining these phases depicts the dynamics of tissue growth and nutrient prioritization during postnatal animal growth. In fact, commercial swine diets are formulated in phases to optimize the FE and accommodate tissue-specific energy needs throughout a pig’s life [3]. Therefore, understanding the physiological changes that facilitate efficient tissue growth is imperative to continue improving feed conversion in commercial swine operations. However, the metabolic characteristics of muscle in growing pigs have not been defined. The objective of this study was to determine the metabolic characteristics of muscles with metabolisms ranging from inherently glycolytic to oxidative in pigs from weaning to market.

## 2. Materials and Methods

### 2.1. Animal Harvest and Sample Collection

The protocol used in this experiment was approved by the Kansas State University Institutional Animal Care and Use Committee (protocol 4485.10, approval date: 25 June 2021). The study utilized castrated male pigs (DNA 600 × 241; DNA Genetics, Columbus, NE, USA) that were housed at the Kansas State University Swine Teaching and Research Center in Manhattan, KS. The nursery pens (5 ft. × 5 ft.) and finisher pens (10 ft. × 8 ft.) allotted 5 ft.^2^ and 8 ft.^2^ per pig, respectively, to maintain a consistent pen density and size for each age group. Pigs were provided with ab libitum access to feed and water with each pen containing a single, dry self-feeder and nipple waterer. Prior to harvest, pigs consumed corn–soybean meal-based diets typical of those fed in commercial production that were formulated to meet or exceed the National Research Council [14] estimates for nutrient requirement for pigs of the given weight range. In order to meet the nutrient requirements for these growing pigs, phase-feeding was implemented to ensure optimal growth. Body weights were collected the day before slaughter. At 20 (weaning), 53, 87, 120, and 180 days of age, the pigs were transported to the KSU Meat Lab and harvested. The days of age were determined based on the feed phasing system established at KSU, which mimics commercial production. The phases meet specific dietary requirements, specifically lysine specifications, correlating to the pig’s current stage of growth. Five barrows of the average group weight were selected at the end of each feeding phase [15]. Samples from the *longissimus dorsi* (LD), *latissimus dorsi* (LAT), and *masseter* (MS) were collected within fifteen minutes after exsanguination. Samples were divided and snap frozen in liquid nitrogen, and frozen for histology or used to isolate mitochondria. Samples snap frozen in liquid nitrogen were stored at −80 °C until further analysis. Histology samples were submerged in plastic molds containing Optimal Cutting Media (Fisher Scientific, Hampton, NH, USA), frozen in liquid nitrogen-cooled isopentane, and stored at −80 °C. Mitochondria were immediately isolated for metabolite tracing.

### 2.2. Backfat Measurement

To gain insight into adipose tissue growth, five barrows (DNA 600 × 241; DNA Genetics, Columbus, NE, USA) of corresponding ages (25 d, 58 d, 92 d, 130 d, and 180 d) were ultrasonically measured for backfat. To maintain consistency, these pigs were raised in the same environmental conditions and followed the same phase-feeding diets as the pigs in the rest of this study, as outlined in Section 2.1. Backfat thickness (BF) was measured using a Renco Lean-Meater Digital Backfat Indicator (Renco Corporation, Golden Valley, MN, USA) from an alternate group of pigs. To determine the correct location for the BF measurement, the last rib was found and an oil spot 65 mm below the spine was marked. Each barrow was measured twice, one measurement for each side of the loin and averaged together.

### 2.3. Histochemistry

Five-micron thick sections were cut using a 550 HM micron (ThermoFisher Scientific, Waltham, MA, USA) and placed on saline-coated slides. The slides were submerged in 1% hematoxylin (Fisher Scientific, Hampton, NH, USA) for 3 min and rinsed in running deionized water. The slides were submerged in 50% eosin (Fisher Scientific, Hampton, NH, USA) for 1 s and rinsed with deionized water. The slides were submerged in increasing concentrations of ethanol, rinsed in xylene for 7 s, dried with a Kimwipe (Kimberly-Clark Professional Kimtech Science, Fisher Scientific, Hampton, NH, USA) and mounted with Epredia™ Shandon-Mount (Fisher Scientific, Hampton, NH, USA). At a 10× magnification, six images were taken per sample with a total of 120 muscle fibers quantified per muscle using a Nikon Eclipse Ti2 inverted microscope (Nikon, Melville, NY, USA) and analyzed with Nikon-Elements software. The cross-sectional areas (µm^2^) from the six images were averaged to obtain a single value per sample for statistical analysis.

### 2.4. Mitochondria Isolation

Mitochondria were isolated using the differential centrifugation method as described in [16] (pp. C355–C356) and [17] (p. 2) with minor modifications. Briefly, muscles were finely minced with scissors at a ratio of 1.5:10 (wt/vol) in ice-cold homogenization buffer (pH 7.4) containing 100 mM sucrose, 180 mM KCl, 50 mM Tris, 5 mM MgCl_2_, 10 mM EDTA, and 1 mM K-ATP. Protease from *Bacillus licheniformis* (Cat #. 9014-01-1, Sigma Aldrich, St. Louis, MO, USA) was added to the tissue suspension at a final concentration of 0.4 mg/mL and homogenized using a Potter-Elvehjem tissue homogenizer system (Glas-Col, Terre Haute, IN, USA). The homogenates were diluted with homogenization buffer to ~40 mL/g of tissue and filtered through cheesecloth. The filtered homogenates were centrifuged at 2000× *g* for 10 min, and the supernatants were filtered again through cheesecloth. The filtered supernatants were centrifuged at 8000× *g* for 10 min. The mitochondrial pellets were resuspended in a modified KHEB buffer (pH 7.2) containing 120 mM KCl, 0.2 mM KH_2_PO_4_, 1 mM HEPES, and 0.5 mM EGTA. The mitochondrial protein concentration was determined by Thermo Scientific™ Pierce™ BCA Protein Assay Kit (Pierce, Rockford, IL, USA) according to the manufacturer’s instructions. Mitochondrial enrichment was confirmed using MS and LD mitochondria through Western blotting using an anti-SDHA antibody (Cat # NBP#-13522, Novus Biologicals, Centennial, CO, USA), as shown in Figure S3. Western blotting conditions are described in Section 2.7 below.

### 2.5. In Vitro [^13^C_3_]-Pyruvate and [^13^C_5_]-Glutamate Tracing in Isolated Mitochondria

In a microcentrifuge tube, 3 mg of isolated mitochondria were added to 1 mL of KHEB containing 1 mM malic acid, 5 mM ADP, and 1 mM sodium pyruvate-^13^C_3_ (Sigma Aldrich, St. Louis, MO, USA) or 1 mM glutamate-^13^C_5_ (Cambridge Isotope Laboratories, Inc., Tewksbury, MA, USA). Unlabeled control reactions contained 1 mM sodium pyruvate or 1 mM glutamate. After incubation at 37 °C on a rocker at 40 rpm for 45 min, the samples were mixed with 2 M perchloric acid, incubated on ice for 15 min, and centrifuged at 12,000× *g* for 5 min. The supernatant was immediately transferred to new tubes, neutralized with 3 M KOH, and stored at −80 °C until further analysis [17,18].

### 2.6. Mass Spectrometry of TCA Metabolites

Samples and TCA intermediate standards were prepared for gas chromatography–mass spectrometry (GM-MS) using the deproteinization and derivatization method described in [17] (pp. 2–3). Briefly, 5 M hydroxylamine hydrochloride was added to the samples followed by the addition of 4 M KOH. At a basic pH, the samples were sonicated and then acidified with 6 N HCl. Ethyl acetate was added to samples to extract the organic phase of the supernatant after centrifugation. The samples were evaporated to dryness under compressed N_2_ at 40 °C. Once dried, equal amounts of N-tert-butyldimethylsilyl-N-methyltrifluoroacetamide (MTBSTFA) and ethyl acetate were added to form the tertiary-butyldimethylsilyl (tBDMS) derivatives during a 1 h incubation period at 65 °C. The samples were separated using gas chromatography (Agilent 6890N; Agilent, Santa Clara, CA, USA) on a J&W HP-5 ms GC Column (HP-5MS, 30 m, 0.25 mm, 0.25 µm film; Agilent Technologies), as previously described in [19] (pp. 870–871) with minor modifications. Intermediate ions were evaluated based on their mass-to-charge ratio (*m/z*) using the single ion monitoring method with a mass spectrophotometer (Agilent 5973N; Agilent, Santa Clara, CA, USA). The ions monitored included the following: pyruvate (274.10); succinate (289.10); fumarate (287.10); malate (419.20); oxaloacetate (432.10); α-ketoglutarate (446.20); and citrate (591.40). The mole percent excess (MPE) was determined through the peak area and was calculated as (M + i/M + 0) × 100, where M + i was defined as the isotopic enrichment of a molecule.

### 2.7. Western Blotting

Muscle samples were pulverized in liquid nitrogen, homogenized in ice-cold IntactProtein™ lysis buffer (GenuIN Biotech, Blacksburg, VA, USA) according to manufacturer’s instructions, and centrifuged at 13,000× *g* for 10 min at 4 °C. The supernatants were collected and protein concentration was determined with a BCA Protein Assay Kit, as mentioned previously. The samples were diluted in 2X Laemmli sample buffer containing 2-mercaptoethanol, sodium dodecyl sulfate, glycerol, tris-hydrochloride, bromophenol blue, protease inhibitor mini tablets (Cat #. PIA32955, Fisher Scientific, Hampton, NH, USA), and phosphatase inhibitor mini tablets (Cat #. A32957, Fisher Scientific, Hampton, NH, USA). Equal amounts of protein were separated using SDS-PAGE and transferred to PVDF or nitrocellulose membranes. The membranes were blocked with OneBlock blocking buffer (Prometheus Protein Biology Products, Genesee Scientific, San Diego, CA, USA) and incubated with the corresponding primary antibody overnight at 4 °C. The secondary antibodies used included Goat anti-Rabbit IgG (H + L) Highly Cross-Adsorbed Secondary Antibody, Alexa Fluor™ Plus 647, Invitrogen™ (Cat# PIA32733, Fisher Scientific, Hampton, NH, USA), and goat anti-rabbit IgG antibody (H + L) HRP conjugate (Cat# AP307P, Sigma Aldrich, St. Louis, MO, USA). The enzymes used for the Western blot analysis were as follows (primary antibody, primary antibody concentration, secondary antibody, secondary antibody concentration, blocking time and temperature, and image detection): lactate dehydrogenase (anti-LDHα (Cat #: NBPI-48336SS, Novus Biologicals, Centennial, CO, USA), 1:2000, goat anti-rabbit Alexa Fluor™ Plus 647, 1:10,000, 30 min at room temperature, fluorescence); glutamate dehydrogenase (anti-GDH (Cat #: PIPA569381, Fisher Scientific, Hampton, NH, USA), 1:1000, goat anti-rabbit HRP, 1:9000, 2 h at room temperature, chemiluminescence); glutamic-oxaloacetic transaminase 2 (anti-GOT2 (Cat# NBP2-32241, Novus Biologicals, Centennial, CO, USA), 1:1000, goat anti-rabbit Alexa Fluor™ Plus 647, 1:10,000, 1 h at room temperature, fluorescence); pyruvate dehydrogenase alpha 1 (anti-PDHA1 (Cat# AV48137, Sigma Aldrich, St. Louis, MO, USA), 1:1000, goat anti-rabbit HRP, 1:30,000, 2 h at room temperature, chemiluminescence); mitochondrial citrate carrier (anti-SLC25A1 (Cat# NBPI-31851, Novus Biologicals, Centennial, CO, USA), 1:1000, goat anti-rabbit HRP, 1:10,000, 2 h at room temperature, chemiluminescence); citrate synthase (anti-CS (Cat# TA308265, OriGene, Rockville, MD, USA), 1:1000, goat anti-rabbit HRP, 1:10,000, 2 h at room temperature, chemiluminescence); pyruvate carboxylase (anti-PC (Cat# NBPI-49536SS, Novus Biologicals, Centennial, CO, USA), 1:1000, goat anti-rabbit HRP, 1:9000, 2 h at room temperature, chemiluminescence); and succinate dehydrogenase (anti-SDHA antibody (Cat # NBP#-13522, Novus Biologicals), 1:1000, goat anti-rabbit Alexa Fluor™ Plus 555, 1:8000, 30 min at room temperature, fluorescence). All antibodies were diluted in OneBlock blocking buffer (Prometheus Protein Biology Products, Genesee Scientific, San Diego, CA, USA). Total protein abundance was measured with the Invitrogen ™ No-Stain™ Protein labeling reagent (ThermoFisher Scientific, Walham, MA, USA) following the manufacturer’s instructions. Prior to imaging, ProSignal Pico ECL reagent (Prometheus Protein Biology, Genesee Scientific, San Diego, CA, USA) was added to the membranes; they were imaged with chemiluminescence for 45 s and then drained. Membranes were imaged with the IBright Imaging System (ThermoFisher Scientific, Waltham, MA, USA). Images were analyzed using Thermo Fisher iBright Analysis Software and normalized to the total protein abundance.

### 2.8. Statistics Section

The data were analyzed as a 3 × 5 factorial design using a mixed-effects model with fixed effects of muscle, age, and their interaction, and a random effect of pig. Data were considered significant when *p* ≤ 0.05 and the means were separated by pairwise comparisons using Tukey’s HSD. Main effect means (muscle or age) are reported when the interaction was not significant (muscle × age). Data are presented as the least squares means +/− standard error. All data were analyzed using R (version 4.2.1, Indianapolis, IN, USA). Western blot data were log transformed to normalize the residuals. Data are presented as the back-transformed values. Pearson’s correlation coefficient was calculated between the normalized abundance of GDH and cross-sectional area as well as between the normalized abundance of GOT2 and cross-sectional area using R (version 4.2.1, Indianapolis, IN, USA).

## 3. Results

A growth model was used to evaluate the changes in skeletal muscle metabolism from weaning to market. Ages were selected based on the completion of each dietary phase fed at the Kansas State University Swine Unit, and pigs were harvested prior to moving on to the next phase of feeding. Within each age group, three muscles with different inherent metabolic profiles were assessed to define the metabolic profile of skeletal muscle during postnatal muscle growth. Samples were collected from the *longissimus dorsi* (LD, glycolytic muscle), *latissimus dorsi* (LAT, mixed muscle), and *masseter* (MS, oxidative muscle) at 20, 53, 87, 120, and 180 days of age from DNA 600 × 241 (DNA Genetics, Columbus, NE, USA) castrated male pigs that weighed an average of 5.7, 20.8, 42.2, 83.4, and 130.5 kg, respectively.

### 3.1. Muscle and Fat Growth Characterization

**Figure 1:**
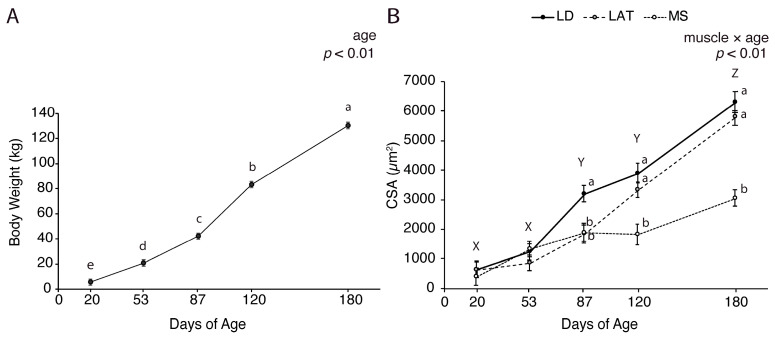
Growth characteristics of pigs at each age point. (**A**) Body weight of pigs at 20, 53, 87, 120, and 180 d. (**B**) Muscle cross-sectional area (CSA) of LD, LAT, and LD muscles collected from pigs at each age point. Data are shown as means ± SE. Five barrows per age group (n = 5). Means lacking a common letter (a, b, c, d, e) differed within a time point (*p* < 0.05). When the interaction was significant (muscle × age), means lacking a common letter (a, b) differ within a time point and (X, Y, Z) differ between ages.

The body weight and muscle cross-sectional area were measured to evaluate the overall weight gain and muscle hypertrophy at 20, 53, 87, 120, and 180 d. The body weight (Figure 1A; *p* < 0.01).) increased at each age point from 20 to 180 d. The cross-sectional area (CSA) was assessed to determine the fiber size at each age. The muscle fiber CSA in LD and LAT muscles increased at each age point after 53 d, while the fibers in MS muscles only increased in CSA at 180 d (Figure 1B and Figure S1; *p* < 0.01). Of note, the MS fiber size appeared to stall between 87 and 120 d and 120 and 180 d, which is a period of rapid hypertrophy in LAT and LD muscles (Figure 1B and Figure S1; *p* < 0.01). These data show that intensive muscle hypertrophy begins at 53 d in LD and 87 d in LAT, while minimal hypertrophy occurs in MS muscles.

Backfat thickness was measured in a different group of barrows that were raised under the same conditions as barrows evaluated herein to provide general insights into adipose tissue growth characteristics at the corresponding time points. The backfat thickness increased at each age point, and there was a 2-fold increase in backfat from 130 to 180 d (Figure S2; *p* < 0.01), indicating that the beginning of intensive adipose tissue growth occurs around 130 d in DNA 600 × DNA 241 (DNA Genetics, Columbus, NE, USA) barrows reared under commercial conditions.

### 3.2. Enzymes Involved in Pyruvate Metabolism Are Variable with Age

To assess skeletal muscle metabolism during postnatal growth, we selected several enzymes that can provide insights into metabolic changes. For example, to determine if glucose fate changes during postnatal growth, we assessed lactate dehydrogenase alpha (LDHα), pyruvate dehydrogenase alpha 1 (PDHα1), and pyruvate carboxylase (PC) levels. These enzymes all convert pyruvate to different intermediates that proceed through varying metabolic routes. Highly glycolytic muscle upregulates LDHα to increase glycolytic flux by converting pyruvate to lactate, which is then shuttled from the muscle into the Cori cycle where lactate is converted to glucose through gluconeogenesis in the liver. Alternatively, pyruvate can enter the TCA cycle through the PDH complex or PC. Although pyruvate largely enters the TCA cycle through the PDH complex, PC catalyzes the carboxylation of pyruvate to oxaloacetate, which is an alternative entry point that does not oxidize pyruvate [20].

**Figure 2:**
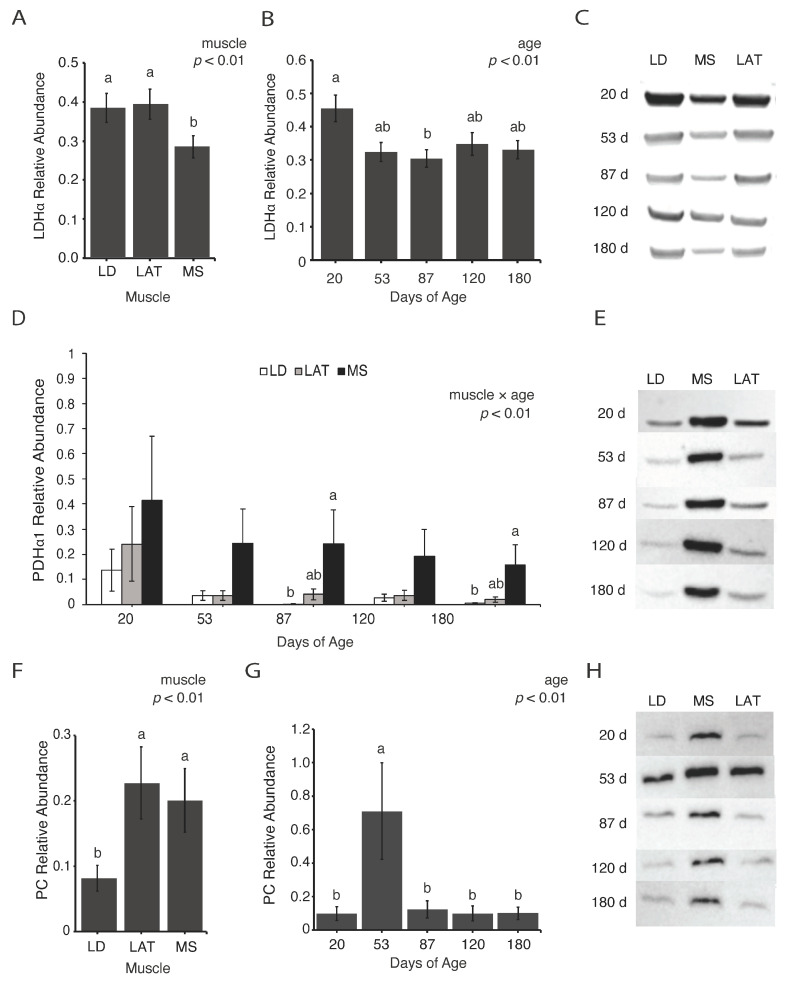
Relative abundance of enzymes involved in pyruvate metabolism. (**A**,**B**) Relative abundance of lactate dehydrogenase alpha (LDHα) in each (**A**) muscle and (**B**) age group. (**C**) Representative images of Western blots quantified in (**A**,**B**). (**D**) Relative abundance of pyruvate dehydrogenase alpha 1 (PDHα1). (**E**) Representative images of Western blots quantified in (**D**). (**F**–**H**) Relative abundance of pyruvate carboxylase (PC) in each (**F**) muscle and (**G**) age group. (**H**) Representative images of Western blots quantified in (**F**,**G**). Data are shown as means ± SE. Five barrows per age group (n = 5). Means lacking a common letter differed (a, b) within a time point (*p* < 0.05).

The abundance of LDHα was lowest in MS compared to LAT and LD muscles at all ages (Figure 2A,C; *p* < 0.01). Additionally, LDHα decreased from 20 to 87 d in all muscles (Figure 2B,C; *p* < 0.01), which may be attributed to the muscles being more glycolytic at weaning from the lactose in their milk-based diet [21]. To assess the adjustments in mitochondrial pyruvate metabolism, the abundance of PDHα1 and PC were determined. At 87 and 180 d, the PDHα1 abundance increased in MS compared to LD muscles (Figure 2D,E; *p* < 0.01). The abundance of PC was highest in LAT and MS compared to LD muscles at all time points (Figure 2F,H; *p* < 0.01). In all muscles, the PC abundance increased at 53 d compared to all the other time points (Figure 2G,H; *p* < 0.01). These data suggest that pyruvate metabolism is dynamic during postnatal growth.

### 3.3. Enzymes Involved in Citrate Metabolism Vary with Age

Pyruvate has many potential fates after entering the TCA cycle, which has implications on efficient metabolite utilization. While a comprehensive assessment of all mitochondria pathways is technically challenging, investigations into specific pathways provides insights into the potential adaptations that mitochondria employ during postnatal muscle hypertrophy. For example, citrate efflux from mitochondria is associated with modulating the glycolytic flux through different mechanisms. Therefore, the abundance of citrate synthase (CS) and mitochondrial citrate carrier (MCC) were assessed. Oxaloacetate and acetyl-CoA are condensed by CS to produce citrate, and citrate can be shuttled out of mitochondria through MCC.

**Figure 3:**
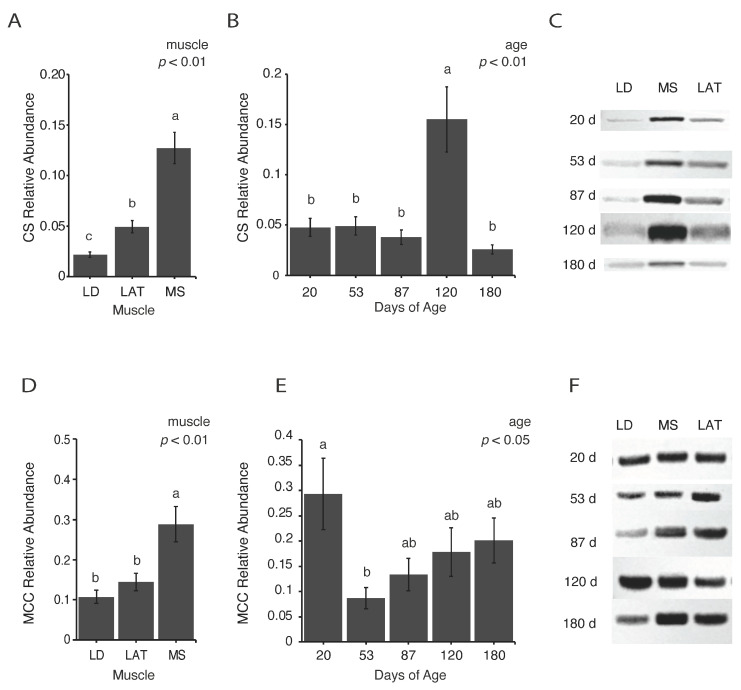
Relative abundance of enzymes involved in citrate metabolism. (**A**,**B**) Relative abundance of citrate synthase (CS) in each (**A**) muscle and (**B**) age group. (**C**) Representative images of Western blots quantified in (**A**,**B**). (**D**,**E**) Relative abundance of mitochondria citrate carrier (MCC) in each (**D**) muscle and (**E**) age group. (**F**) Representative images of Western blots quantified in (**D**,**E**). Data are shown as means ± SE. Five barrows per age group (n = 5). Means lacking a common letter (a, b, c) differed within a time point (*p* < 0.05).

At all ages, the CS abundance was highest in MS and lowest in LD with the abundance in LAT muscles being intermediate (Figure 3A,C; *p* < 0.01). The abundance of CS in all muscles increased at 120 d compared to all the other ages (Figure 3B,C; *p* < 0.01). Citrate can continue through the TCA cycle to be oxidized to meet cellular ATP demands or be shuttled out of mitochondria through MCC. The abundance of MCC was assessed as an indicator of changes in citrate efflux. At all ages, the MCC abundance was highest in MS compared to LAT and LD muscles (Figure 3D,F; *p* < 0.01). In all muscles, MCC decreased at 53 d compared to 20 d (Figure 3E,F; *p* < 0.05). These data indicate that citrate flux through the TCA cycle changes during growth in all muscles investigated.

### 3.4. Abundance of Mitochondrial Enzymes Involved in Amino Acid Metabolism Differs Based on Inherent Muscle Metabolism

**Figure 4:**
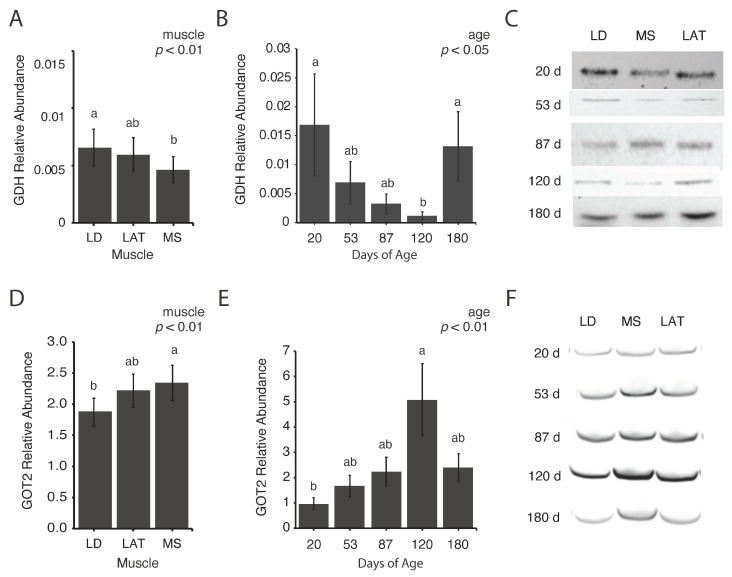
Relative abundance of enzymes involved in amino acid metabolism. (**A**,**B**) Relative abundance of glutamate dehydrogenase (GDH) in each (**A**) muscle and (**B**) age group. (**C**) Representative images of Western blots quantified in (**A**,**B**). (**D**,**E**) Relative abundance of glutamic-oxaloacetic transaminase (GOT2) in each (**D**) muscle and (**E**) age group. (**F**) Representative images of Western blots quantified in (**D**,**E**). Data are shown as means ± SE. Five barrows per age group (n = 5). Means lacking a common letter (a, b) differed within a time point (*p* < 0.05).

To determine if amino acid metabolism changes in muscles during postnatal growth, glutamate dehydrogenase (GDH) and glutamic-oxaloacetic transaminase 2 (GOT2) abundances were assessed. These two enzymes were selected because they catalyze reactions involving different TCA intermediates and amino acids, and therefore provide insights into different aspects of amino acid metabolism. The abundance of GDH, which catalyzes the reversible conversion of glutamate to α-ketoglutarate, increased in LD compared to MS muscles (Figure 4A,C; *p* < 0.01) and decreased at 120 d compared to 20 and 180 d (Figure 4B,C; *p* < 0.05). The abundance of GOT2, which synthesizes aspartate from oxaloacetate and glutamate, increased in MS compared to LD muscles (Figure 4D,F; *p* < 0.01) and increased at 120 d compared to 20 d (Figure 4E,F; *p* < 0.01). Further, the correlation coefficients of GDH and GOT2 abundances with muscle CSA were 0.59 and −0.44, respectively. Although these enzymes are both involved in amino acid metabolism, muscles with inherently divergent metabolic profiles differ in the abundance of these enzymes.

### 3.5. Assessment of Pyruvate-Derived TCA Intermediate Metabolism in the TCA Cycle

**Figure 5:**
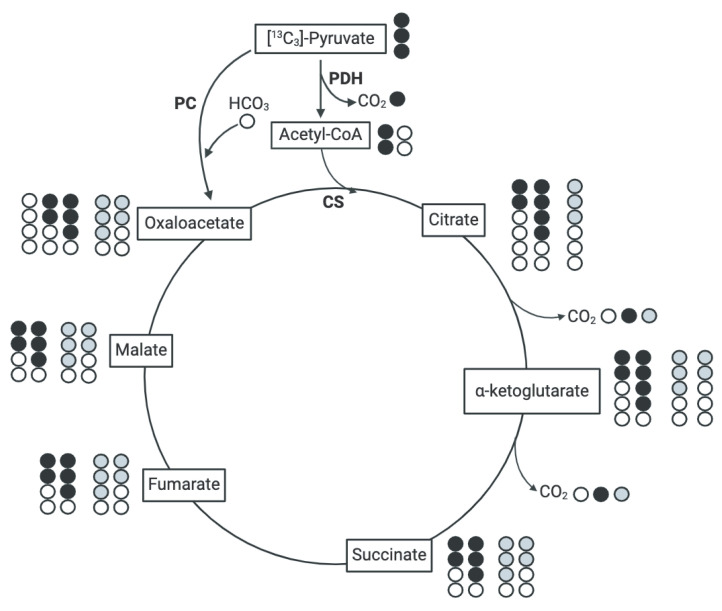
Schematic of potential labeling patterns of TCA intermediates after isolated mitochondria were incubated with [^13^C_3_]-pyruvate. Black circles are ^13^C derived from [^13^C_3_]-pyruvate that entered the TCA cycle through PDH. Grey circles indicate ^13^C derived from [^13^C_3_]-pyruvate that entered the TCA cycle through PC. White circles represent unlabeled carbon atoms (^12^C).

To establish a more comprehensive understanding of how muscles allocate and utilize nutrients during postnatal muscle growth, we implemented an in vitro stable isotope tracing model adapted from [17] (pp. 2–3), [22] (p. 851), and [23] (pp. 167–169) to uncover changes in pyruvate-derived TCA intermediate flux. After incubation of isolated mitochondria with [^13^C_3_]-pyruvate and unlabeled malate, the isotopomer enrichment patterns of TCA intermediates were analyzed (Figure 5). Briefly, the number of labeled carbons (or ^13^C) in TCA intermediates derived from [^13^C_3_]-pyruvate can be detected using GC-MS. The enrichment patterns depict the number of ^13^C in each intermediate that originated from [^13^C_3_]-pyruvate. Values for M + i represent the isotopomer proportions of a given metabolite, and therefore do not provide information on the total amount of metabolites in each sample but rather the contribution of pyruvate to the TCA cycle. For example, M + 2 citate is indicative of two ^13^C in the citrate molecule originating from the labeled pyruvate molecule (Figure 5). Labeling patterns can be traced throughout subsequent TCA reactions [20], which are depicted theoretically as black or grey circles in Figure 5. Labeling patterns can be traced throughout subsequent TCA reactions [20], which are depicted theoretically as black or grey circles in Figure 5.

**Figure 6:**
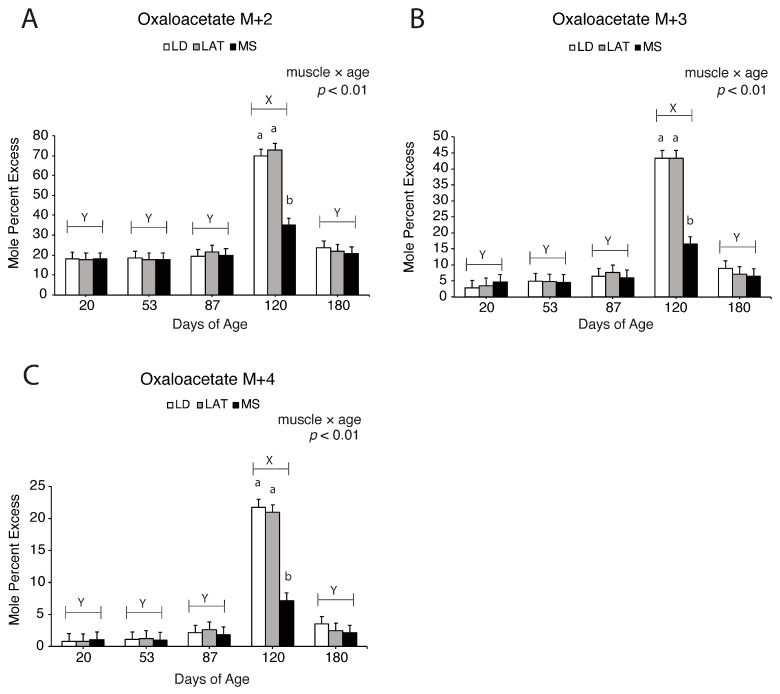
[^13^C_3_]-pyruvate derived isotopomer enrichment of oxaloacetate in isolated mitochondria. (**A**–**C**) Enrichment of (**A**) M + 2, (**B**) M + 3, and (**C**) M + 4 oxaloacetate in LD, LAT, and LD mitochondria isolated from pigs at each age point. Data are shown as means ± SE. Mitochondria were isolated from LD, LAT, and MS muscles of five barrows per age group (n = 5). Means lacking a common letter (a, b) differed within a time point (*p* < 0.05). When the interaction was significant (muscle × age), means lacking a common letter (a, b) differ within a time point and (X, Y) differ between ages.

**Figure 7:**
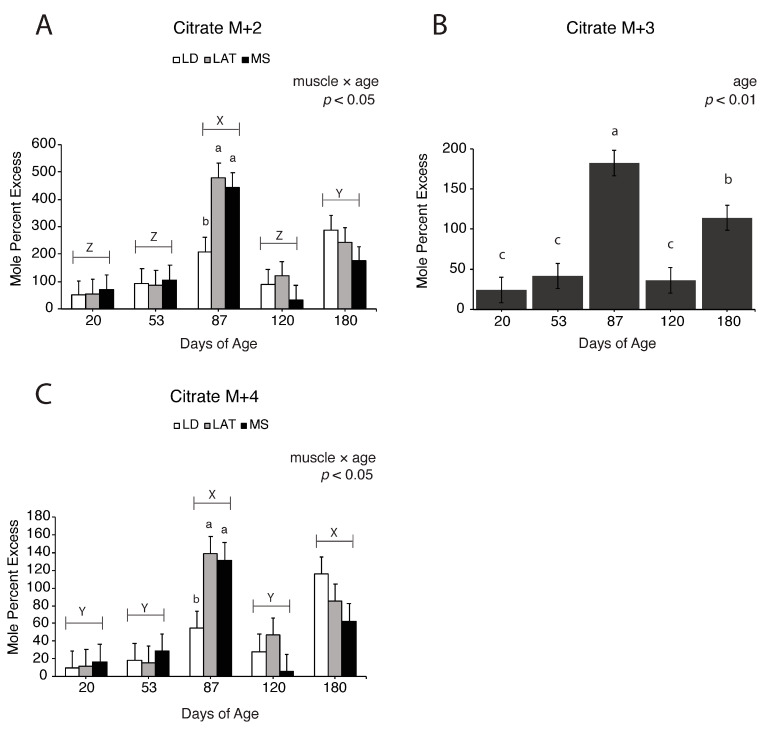
[^13^C_3_]-pyruvate derived isotopomer enrichments of citrate in isolated mitochondria. (**A**–**C**) Enrichment of (**A**) M + 2, (**B**) M + 3, and (**C**) M + 4 citrate in LD, LAT, and LD mitochondria isolated from pigs at each age point. Data are shown as means ± SE. Mitochondria were isolated from LD, LAT, and MS muscles of five barrows per age group (n = 5). Means lacking a common letter (a, b, c) differed within a time point (*p* < 0.05). When the interaction was significant (muscle × age), means lacking a common letter (a, b, c) differ within a time point and (X, Y, Z) differ between ages.

**Figure 8:**
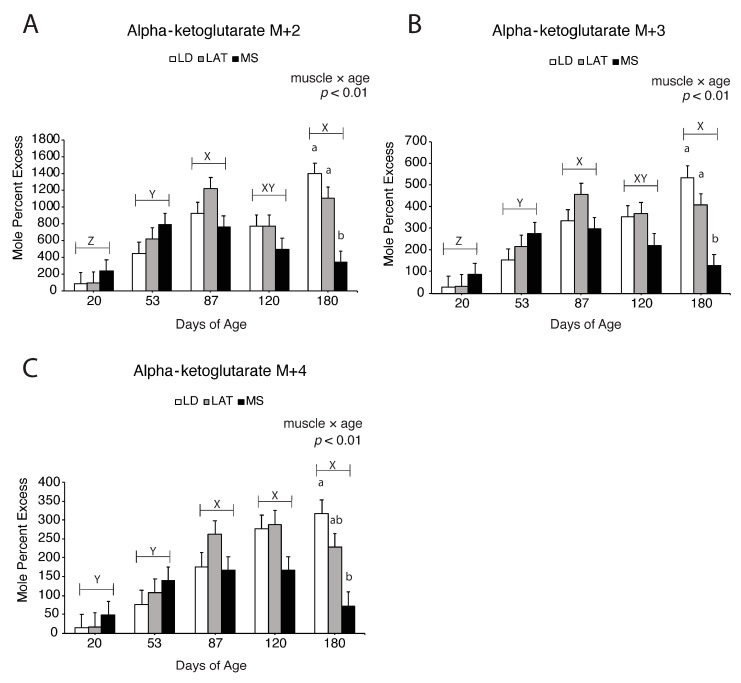
[^13^C_3_]-pyruvate derived isotopomer enrichments of α-ketoglutarate in isolated mitochondria. (**A**–**C**) Enrichment of (**A**) M + 2, (**B**) M + 3, and (**C**) M + 4 α-ketoglutarate in LD, LAT, and LD mitochondria isolated from pigs at each age point. Data are shown as means ± SE. Mitochondria were isolated from LD, LAT, and MS muscles of five barrows per age group (n = 5). Means lacking a common letter (a, b) differed within a time point (*p* < 0.05). When the interaction was significant (muscle × age), means lacking a common letter (a, b) differ within a time point and (X, Y, Z) differ between ages.

Enrichment fractions of oxaloacetate, citrate, and α-ketoglutarate derived from [^13^C_3_]-pyruvate are reported as mole percent excess (MPE). For example, M + 2 oxaloacetate is (M + 2 oxaloacetate/M+0 oxaloacetate) × 100. Oxaloacetate M + 2, M + 3, and M + 4 increased in LD and LAT mitochondria compared to MS mitochondria at 120 d (Figure 6A–C; *p* < 0.01), which suggests that pyruvate-derived oxaloacetate increased in LD and LAT mitochondria at this time. Citrate M + 2 and M + 4 increased in LAT and MS mitochondria compared to LD mitochondria at 87 d (Figure 7A,C; *p* < 0.05). Regardless of muscle, citrate M + 3 increased at 87 d compared to 20, 53, and 120 d, while 180 d showed intermediate values (Figure 7B; *p* < 0.01). Alpha-ketoglutarate M + 2 (Figure 8A; *p* < 0.01) and M + 3 (Figure 8B; *p* < 0.01) increased in LD and LAT mitochondria compared to MS mitochondria at 180 d, while α-ketoglutarate M + 4 increased in LD mitochondria compared to MS mitochondria at 180 d (Figure 8C; *p* < 0.01). These data indicate that TCA cycle metabolite flux changes during postnatal growth.

### 3.6. Assessment of Glutamate Entry into the TCA Cycle

**Figure 9:**
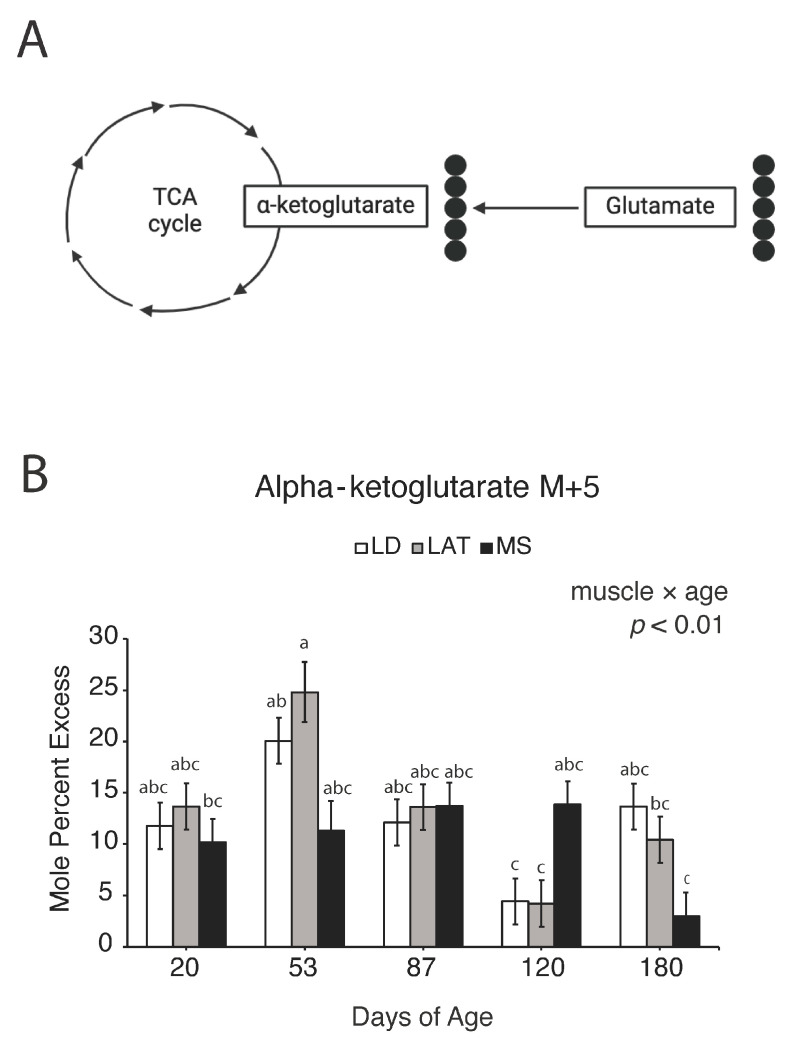
(**A**) Schematic of potential labeling pattern of α-ketoglutarate from glutamate after isolated mitochondria were incubated with [^13^C_5_]-glutamate. Black circles are ^13^C derived from [^13^C_5_]-glutamate that entered the TCA cycle as α-ketoglutarate. (**B**) [^13^C_5_]-glutamate-derived isotopomer enrichments of α-ketoglutarate in isolated mitochondria. Enrichment of (B) M + 5 α-ketoglutarate in LD, LAT, and LD mitochondria isolated from pigs at each age point. Data are shown as means ± SE. Mitochondria were isolated from LD, LAT, and MS muscles of five barrows per age group (n = 5). Means lacking a common letter (a, b, c) differed across the age and muscle groups (*p* < 0.05).

Glutamate is a central amino acid involved in many transamination reactions integrated into the TCA cycle that are essential for amino acid metabolism [24]. To understand the changes in glutamate metabolism during muscle hypertrophy, we implemented the in vitro stable isotope tracing model as described in Section 3.5 but with [^13^C_5_]-glutamate and unlabeled malate (Figure 9A). After incubation, the isotopomer enrichment pattern of M + 5 α-ketoglutarate was determined, which is indicative of glutamate-derived α-ketoglutarate levels. In LD and LAT mitochondria, α-ketoglutarate M + 5 increased at 53 d compared to 120 d (Figure 9B; *p* < 0.01). At 53 d, LD and LAT mitochondria had an increase in α-ketoglutarate M + 5 compared to MS mitochondria at 180 d (Figure 9B; *p* < 0.01).

## 4. Discussion

The nutrient distribution to different tissues in a growing animal changes from birth to maturity and can be divided into four theoretical phases. After birth, phase I is characterized by growth and nutrient allocation to vital organs, visceral fat, the nervous system, and bone development. The next phase of growth allocates nutrients to support rapid muscle growth whereas the last two phases begin to slow muscle growth and increase fat deposition [9]. Our data identified similar growth trends through an increase in muscle hypertrophy in the LD and LAT from 53 to 180 d (*p* < 0.01). However, our findings did not show muscle hypertrophy slowing between 120 and 180 d in the LD and LAT, which may indicate that the industry has selected for continued and robust hypertrophy throughout finishing or there are changes that are hidden within the 60 days between our final sampling points. In the MS, the muscle fiber CSA only differed at 180 d compared to 20 and 53 d, which shows that minimal hypertrophy occurred in MS. This may be attributed to the smaller fiber size in oxidative muscles compared to glycolytic muscles [25] or the location of the MS muscle in the body.

We measured backfat thickness in a different group of barrows raised in the same conditions as those assessed in this study to gain insights into potential changes in the adipose tissue deposition rate. In the second group of pigs, the backfat thickness increased at all age points but increased 2-fold from 130 to 180 d (*p* < 0.01), which suggests that the adipose tissue deposition rate increases during the last phase of feeding. Although direct comparisons between data collected herein and backfat thickness cannot be made, these supplemental data support the generalized depiction of postnatal tissue growth.

Previous investigations into metabolic changes during postnatal muscle growth have been limited to segments of postnatal growth rather than spanning the scope of weaning to market. For example, [26] reported that LDH activity increased from birth to weaning and then slowed abruptly after 20 days of age but did not assess LDH activity through later phases of growth. While swine genetics have changed since this report, it follows a similar trend to our findings that LDHα decreases after weaning and supports the notion that muscles are more glycolytic prior to weaning while on a milk-based diet. For example, lactose is broken down by lactase into galactose and glucose, which then enters glycolysis. In fact, weanling starter diets include lactose supplementation to support transitioning from sow to self-feeding [21]. At approximately 90 kg, high FE pigs exhibit a decrease in most mitochondrial enzymes and an increase in most glycolytic enzymes compared to low FE pigs [11]. During finishing, studies are focused on different approaches to impact FE and lean muscle growth [27,28,29,30]. Although these findings depict the dynamic nature of muscle, there is a lack of data identifying the metabolic changes that occur in swine muscles from birth to market weight.

The glycolytic end product, pyruvate, can be shuttled from muscle into the TCA cycle or enter the TCA cycle through PDH or PC. When pyruvate is converted to acetyl-CoA by PDH, CO_2_ is produced and represents a loss of a carbon molecule, whereas PC condenses pyruvate and bicarbonate to generate oxaloacetate. While PC abundance and activity is higher in gluconeogenic tissues, PC contributes to metabolite utilization in muscle. In fact, the protein abundance of PC is greater in the *longissimus dorsi* of high-FE pigs compared to low-FE pigs [11]. Further, in vitro supplementation of muscle with different substrates provokes variable PC activity [31,32]. These reports suggest that PC plays an active role in skeletal muscle metabolism, albeit in a subdued manner given that PC-mediated entry cannot compensate for the loss of PDH-mediated entry in muscle [33]. Although PDH is responsible for the majority of pyruvate entry into the TCA cycle, the role of PC in skeletal muscle remains unclear. Therefore, we assessed changes in PC abundance and evaluated potential changes in PC-mediated pyruvate entry into the TCA cycle. In isotope tracing experiments with a fully labeled (M + 3) pyruvate entering the TCA cycle, an increase in M + 2 citrate suggested an increase in pyruvate entering the TCA cycle through PDH [23,31,33,34]. When unlabeled oxaloacetate combines with M + 2 acetyl-CoA, which is derived from PDH, M + 2 citrate is produced (Figure 5, black circles). On the other hand, PC converts pyruvate to oxaloacetate and consumes HCO_3−_, which produces M + 3 oxaloacetate (Figure 5, grey circles). At 87 d, citrate M + 3 increased in all muscles (*p* < 0.01) while citrate M + 2 and citrate M + 4 only increased in LAT and MS mitochondria (*p* < 0.05). These data suggest that at 87 d, LD mitochondria may have a greater reliance on pyruvate entry into the TCA cycle through PC. Although PC did not increase and no changes in PDHα1 abundance were observed in LD muscle at 87 d, it is possible there were changes in the activity of either enzyme. While we attempted to assess PDH phosphorylation several times, we were unsuccessful; however, it is clear in our findings that pyruvate metabolism is altered in muscle through a pig’s life. Future research into the mechanisms and implications of pyruvate entry into the TCA cycle is necessary to understand the complex relationship between PDH and PC in muscle hypertrophy.

Regardless of how pyruvate enters the TCA cycle, CS converts oxaloacetate and acetyl-CoA to citrate. Although the TCA cycle is referred to as a cycle, implying a continuous circle of enzymatic reactions, intermediates can exit the cycle depending on cellular needs. The exit of TCA intermediates, or cataplerosis, can contribute to the synthesis of many molecules and/or initiate signaling events [35]. Citrate can be shuttled out of mitochondria into the cytosol to be converted to oxaloacetate and acetyl-CoA, which has been established as a precursor for histone acetylation to regulate gene expression and fatty acid synthesis [36,37]. Although we have not investigated the role of this signaling pathway in skeletal muscle hypertrophy, we assessed changes in MCC abundance in addition to CS abundance to determine if citrate metabolism and cataplerosis undergo changes during hypertrophy. Our results showed an increase in CS at 120 d compared to all the other time points. Given that we did not find an increase in pyruvate-derived citrate at 120 d, it raises the question of does muscle rely on different metabolites at this time or is pyruvate-derived citrate being shuttled out of the TCA cycle at 120 d? While we did not find changes in the abundance of MCC, which shuttles citrate out of mitochondria, at 120 d, other avenues for citrate exist other than complete oxidation and leaves the answer unclear.

In addition to evaluating the changes in pyruvate and citrate metabolism, we also assessed glutamate entry into the TCA cycle to gain insights into amino acid metabolism. Glutamate is converted to α-ketoglutarate by GDH, which is a reversible reaction that consumes or generates NH_4_^+^ and NADPH. Glutamate can be oxidized fully to produce ATP when needed or converted to other amino acids, such as aspartate and arginine through TCA enzyme-mediated transamination. Conversely, when glutamate is synthesized, it can be used for protein synthesis or as a precursor for several other amino acids, such as alanine, aspartate, and arginine [24]. Our results showed a decrease in GDH at 120 d compared to 20 and 180 d in all muscles as well as a greater abundance of GDH in LD compared to MS muscles. Since the GDH abundance was higher in glycolytic LD muscle compared to oxidative MS muscle, we correlated GDH and GOT2 abundance with muscle CSA to determine if there was an association between the enzymes involved in amino acid metabolism and muscle size. The correlation coefficients of GDH and GOT2 with muscle CSA were 0.59 and −0.44, respectively, which indicates that not all mitochondrial enzymes play the same role in muscle hypertrophy. An increase in mitochondrial enzyme abundance is typically associated with type I/type IIa fibers that have a smaller cross-sectional area. These findings question the role of mitochondria in muscle hypertrophy; however, further investigation is necessary to understand this relationship.

Since the reaction catalyzed by GDH is reversible, it is unclear if the decrease in GDH abundance is associated with a decrease in glutamate oxidation or removal of α-ketoglutarate from the TCA cycle. To gain some insight, we incubated isolated mitochondria with labeled glutamate and unlabeled malate to assess the changes in glutamate entry into the TCA cycle. In LD and LAT mitochondria, glutamate-derived α-ketoglutarate decreased at 120 d compared to 53 d but decreased in MS mitochondria at 180 d compared to LD and LAT mitochondria at 53 d. Although we do not have a clear understanding of the signaling that facilitates the changes in metabolite use, age and inherent metabolic characteristics play a role in cataplerosis and anaplerosis of TCA intermediates that warrant further investigation. While isotopomer labeling patterns are indicative of the enzymatic changes that would facilitate changes in intermediate use, the complex interplay between the regulation of enzyme expression and activity increases the burden of elucidating these molecular mechanisms. Ultimately, it is clear that glutamine and glutamate supplementation facilitates protein synthesis [38,39,40], but further investigation to understand the intricacies of amino acid utilization during postnatal growth is necessary.

In conclusion, these findings show that skeletal muscle metabolism changes with age. However, additional research into the mechanisms that dictate these changes is imperative to translating these findings to improve production performance. First and foremost, investigating the metabolic changes that occur in the skeletal muscle of gilts is necessary to understand sex-driven differences. Due to the technical limitations of this study, barrows were investigated herein; however, gilts and barrows presumably have different metabolic characteristics that warrant investigation. Further, understanding the use of these metabolic pathways in animals with different production performances or distinct carcass compositions would identify pathways that contribute to efficient lean growth. In addition, investigating enzymes that vary in activity during muscle hypertrophy as regulators of nutrient utilization could identify new targets to evaluate or improve performance in the future. Although previous research has established a relationship between skeletal muscle metabolic characteristics and production performance, our current understanding of this relationship is limited by the complex intricacies of metabolite utilization and the dynamics of muscle growth.

## 5. Conclusions

Our findings revealed skeletal muscle metabolism changes during postnatal growth. Although these data do not show causal relationships between specific metabolic changes and muscle hypertrophy, it is clear that metabolic adaptations occur throughout postnatal growth. For example, pyruvate metabolism and entry into the TCA cycle as well as the downstream use of TCA intermediates varies from weaning to market. Our findings also illustrate the complex dynamics between cataplerosis and anaplerosis of TCA intermediates, which have long been overlooked in the muscle physiology of meat-producing animals. In conclusion, these data depict the intricacies of muscle metabolism and highlight potential avenues in mitochondria metabolism that may be necessary for optimal nutrient utilization during lean muscle deposition.

## Data Availability

The raw data supporting the conclusions of this article will be made available by the authors on request.

## Supplementary Materials

The following supporting information can be downloaded at: https://www.mdpi.com/article/10.3390/metabo14070357/s1, Figure S1: Muscle cross-section images from each muscle and age point; Figure S2: Backfat thickness; Figure S3: Mitochondrial enrichment staining.
