# Supplementary material for: Skeletal Muscle Metabolism Is Dynamic during Porcine Postnatal Growth
Source: Metabolites. 2024 Jun 26;14(7):357. doi: 10.3390/metabo14070357 (PMC11279009; doi:10.3390/metabo14070357)
Supplement: Supplementary file 1 [file metabolites-14-00357-s001.zip › metabolites-3019287-SI/Figure S1.pdf]

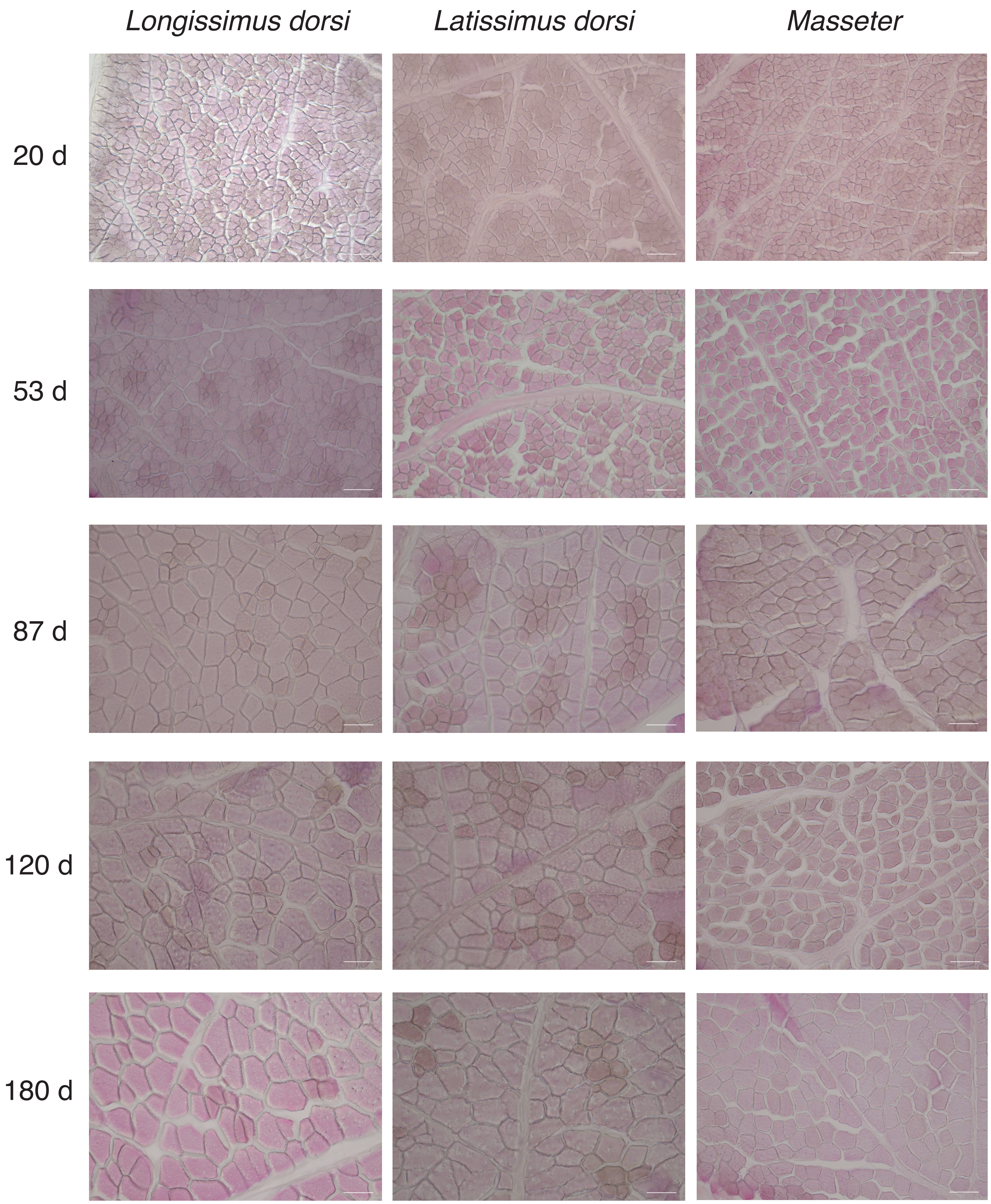

Supplementary Figure S1. Muscle cross-section images from each muscle and age point. Scale bar = 100 μm.
