# Supplementary material for: Skeletal Muscle Metabolism Is Dynamic during Porcine Postnatal Growth
Source: Metabolites. 2024 Jun 26;14(7):357. doi: 10.3390/metabo14070357 (PMC11279009; doi:10.3390/metabo14070357)
Supplement: Supplementary file 1 [file metabolites-14-00357-s001.zip › metabolites-3019287-SI/Figure S2.pdf]

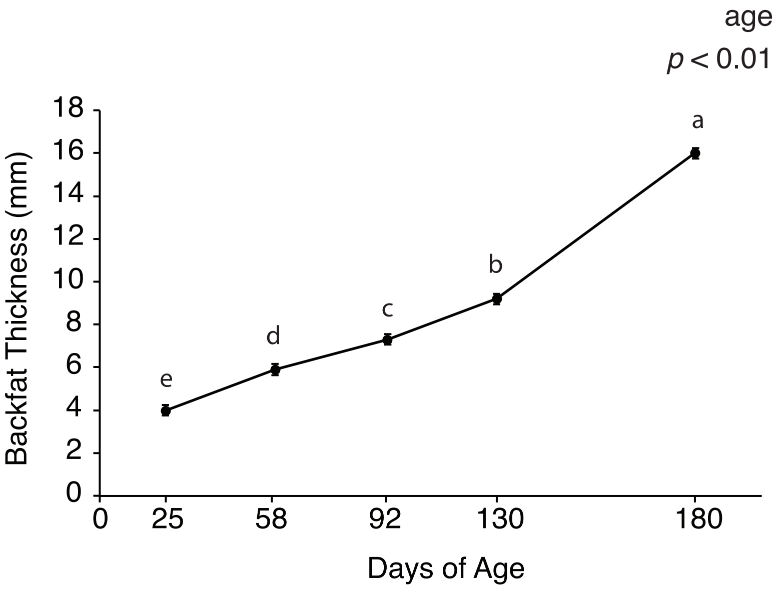

Supplementary Figure S2. Backfat thickness. Barrows raised in the same conditions of those in this study were used to measure backfat thickness at corresponding ages. Data are means ± SE. Five barrows per age group (n=5). Means lacking a common letter (a, c, d, d, e) differ between ages.
