# Supplementary material for: Skeletal Muscle Metabolism Is Dynamic during Porcine Postnatal Growth
Source: Metabolites. 2024 Jun 26;14(7):357. doi: 10.3390/metabo14070357 (PMC11279009; doi:10.3390/metabo14070357)
Supplement: Supplementary file 1 [file metabolites-14-00357-s001.zip › metabolites-3019287-SI/Figure S3.pdf]

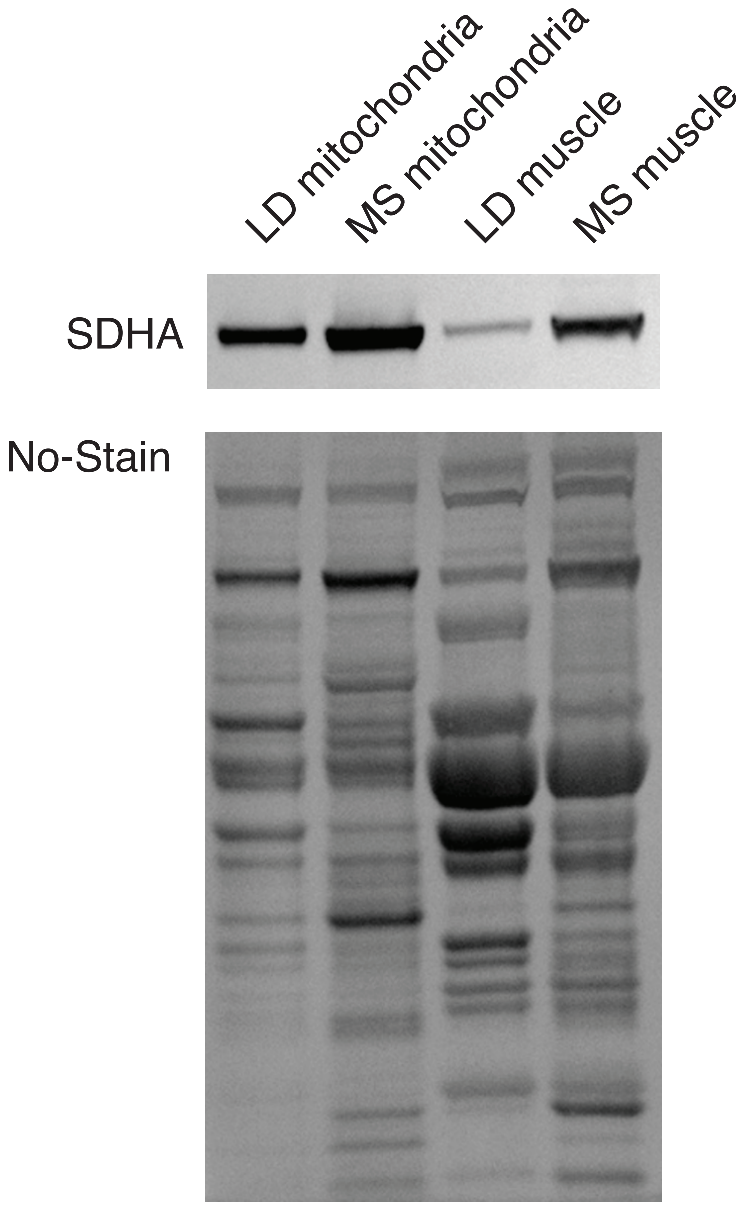

Supplementary Figure S3. Mitochondrial enrichment staining. (Top) Western blot of LD mitochondria (lane 1) and MS mitochondria (lane 2) as well as LD muscle (lane 3) and MS muscle (lane 4) of which mitochondria in lane 1 and 2 were isolated from. (Bottom) Total protein stain using No-Stain Protein Labeling Reagent.
